# The Use of Multifrequency Tympanometry in Ménière’s Disease. Comment on Tsilivigkos et al. Can Multifrequency Tympanometry Be Used in the Diagnosis of Meniere’s Disease? A Systematic Review and Meta-Analysis. *J. Clin. Med.* 2024, *13*, 1476

**DOI:** 10.3390/jcm14020570

**Published:** 2025-01-17

**Authors:** M. A. de Jong, H. G. X. M. Thomeer

**Affiliations:** Otorhinolaryngology and Head and Neck Surgery Department, University Medical Centre Utrecht, PB 85500, 3508 GA Utrecht, The Netherlands; m.dejong@skbwinterswijk.nl

Dear Editor,

With great interest, we have read the review article entitled ‘Can Multifrequency Tympanometry Be Used in the Diagnosis of Meniere’s Disease? A Systematic Review and Meta-Analysis’ [1]. The authors provided an extensive review and meta-analysis addressing the use of multifrequency tympanometry (MFT) as a diagnostic tool in people with Ménière’s disease (MD) or with similar symptoms. The authors discussed both studies that did (or did not) find a significantly greater G-width or Y-width in the multifrequency tympanograms compared to controls.

Based on their careful review, they concluded that MFT could be an important diagnostic test for people with MD. Compared to our study (de Jong et al., 2023 [2]), however, we did not find a significant difference in MFT results between the groups. As described by the authors and is well known concerning the physiologic mechanism of MFT, at the resonance frequency (RF), the admittance (Y) reflecting the acoustic energy that flows into the middle ear consists solely of the conductance (G). Therefore, these two overlap, and the outcome might be comparable. The authors only referred to the G-width (where they found some significant relation to MD), whereas the published articles concerning the Y-width did not show robust evidence supporting the value of MFT for the confirmation of MD. Similarly, the Y-width, as used in our study, did not prove to be significantly increased in MD patients. Based on the literature, it might well be possible that the G-width at 2 kHz in MFT has better diagnostic properties to indicate the presence of endolymphatic hydrops. As mentioned, at resonance frequency (RF), both Y- and G-widths are equal, but not necessarily at 2 kHz. The 2 kHz was chosen as it reflects the annular ligament and cochlear pressure.

The MFT, specifically the G-width, might be a useful tool to diagnose endolymphatic hydrops more than the MD itself, given the mentioned constraints. The explanation as to why we did not find a correlation and others did might be due to the fact that an endolymphatic hydrops can be present in healthy subjects without otovestibular complaints.

Further research is needed to better understand and support theories about pressure changes in MD, as well as the proper definition of actual MD. However, it will not change the fact that there are healthy subjects that demonstrate an increased Y- or G-width because of asymptomatic endolymphatic hydrops not related to MD. This must always be kept in mind. Until we better understand MD, MFT as a diagnostic test should, even in its best form, be used to rule out endolymphatic hydrops or support further evaluation, for example, with MRI hydrops.

Sincerely,

M. A. de Jong

H. G. X. M. Thomeer
